# Supplementary material for: Acquisition of the physiological quality of peanut (Arachis hypogaea L.) seeds during maturation under the influence of the maternal environment
Source: PLoS One. 2021 May 3;16(5):e0250293. doi: 10.1371/journal.pone.0250293 (PMC8092650; doi:10.1371/journal.pone.0250293)
Supplement: S1 Fig — (DOCX) [file pone.0250293.s001.docx]

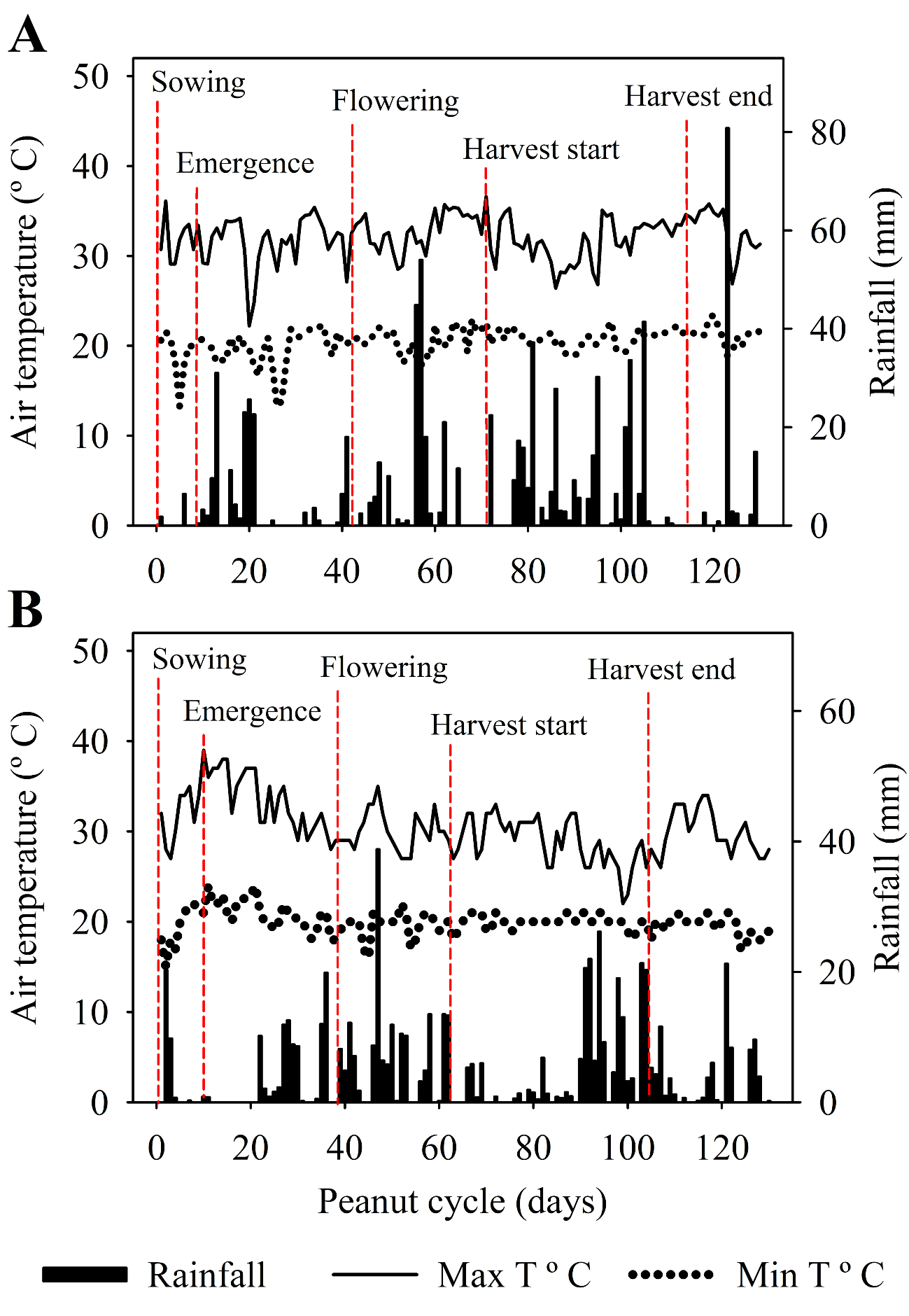


**S1 Fig.** Daily rainfall and maximum and minimum temperatures in the city of Sertãozinho in the State of São Paulo-Brazil during seed production. (A) Represents the data collected during the crop season 2017. (B) Represents the data collected during the crop season 2018.
