# Supplementary material for: Acquisition of the physiological quality of peanut (Arachis hypogaea L.) seeds during maturation under the influence of the maternal environment
Source: PLoS One. 2021 May 3;16(5):e0250293. doi: 10.1371/journal.pone.0250293 (PMC8092650; doi:10.1371/journal.pone.0250293)
Supplement: S1 Table — Crop season 2017. (DOCX) [file pone.0250293.s002.docx]

**S1 Table.** Statistical information of observed data of the variables evaluated in peanut seeds during maturation and late maturarion. Crop season 2017.

| DAF | WC ^1^ | DW | G | DT | t50 | FC |
| --- | --- | --- | --- | --- | --- | --- |
| 28 | 65.8 ± 3.6 a^*^ | 1.8 ± 0.3 d | 12 ± 4.7 d | 6 ± 3.1 d | 25 ± 25.3 c | 6 ± 1.0 e |
| 35 | 46.9 ± 1.5 b | 5.2 ± 0.7 c | 75 ± 1.9 c | 31 ± 3.7 c | 47 ± 28.4 bc | 18 ± 1.2 d |
| 43 | 36.3 ± 0.5 c | 12.1 ± 0.3 b | 84 ± 3.7 c | 46 ± 2.4 b | 156 ± 36.4 a | 41 ± 1.9 c |
| 57 | 32.8 ± 1.3 cd | 14.5 ± 0.1 a | 97 ± 1.9 a | 67 ± 1.2 a | 112 ± 15.7 ab | 59 ± 6.3 b |
| 76 | 28.6 ± 0.4 d | 15.3 ± 0.4 a | 94 ± 4.7 a | 70 ± 1.7 a | 67.8 ± 7.2 bc | 70 ± 4.3 a |
| LSD | 5.6 | 1.3 | 10.9 | 7.8 | 74.6 | 9.3 |
| F *value* | 63.3^**^ | 188.9^**^ | 106.6^**^ | 86.4^**^ | 4.5^**^ | 76.6^**^ |

^1^ WC: water content; DW: dry weight; G: ability of germination; DT: desiccation tolerance; t50: time of 50% germination; FC: first count of germination. ^*^ Averages followed by the same lower case letter in the column do not differ by LSD test at 5% probability. ^**^ significant at 1% by the F test. The means of each variable are accompanied by the standard error.
