# Supplementary material for: Acquisition of the physiological quality of peanut (Arachis hypogaea L.) seeds during maturation under the influence of the maternal environment
Source: PLoS One. 2021 May 3;16(5):e0250293. doi: 10.1371/journal.pone.0250293 (PMC8092650; doi:10.1371/journal.pone.0250293)
Supplement: S2 Table — Crop season 2018. (DOCX) [file pone.0250293.s003.docx]

**S2 Table.** Statistical information of observed data of the variables evaluated in peanut seeds during maturation and late maturarion. Crop season 2018.

| DAF | WC ^1^ | DW | G | DT | t50 | FC |
| --- | --- | --- | --- | --- | --- | --- |
| 28 | 63.2 ± 2.1 a | 1.9 ± 0.2 d | 20 ± 3.7 d | 8 ± 2.9 c | 13 ± 12.7 c | 10 ± 2.6 d |
| 35 | 47.4 ± 1.1 b | 7.3 ± 0.5 c | 56 ± 5.7 c | 26 ± 8.7 b | 118 ± 26.1 ab | 33 ± 7.7 c |
| 43 | 24.7 ± 0.2 c | 10.1 ± 0.6 b | 72 ± 4.3 b | 27 ± 9.6 b | 147 ± 32.5 a | 56 ± 3.6 b |
| 57 | 21.9 ± 0.6 cd | 14.1 ± 0.3 a | 81 ± 2.3 a | 80 ± 1.9 a | 65 ± 11.9 bc | 66 ± 2.1 b |
| 76 | 19.2 ± 0.2 d | 12.9 ± 0.5 a | 87 ± 5.7 a | 83 ± 2.7 a | 73 ± 11.5 bc | 87 ± 5.7 a |
| LSD | 3.3 | 1.3 | 13.6 | 18.4 | 62.9 | 14.6 |
| F *value* | 304.9^**^ | 117.9^**^ | 35.2^**^ | 31.91^**^ | 6.1^**^ | 37.7^**^ |

^1^ WC: water content; DW: dry weight; G: ability of germination; DT: desiccation tolerance; t50: time of 50% germination; FC: first count of germination. ^*^ Averages followed by the same lower case letter in the column do not differ by LSD test at 5% probability. ^**^ significant at 1% by the F test. The means of each variable are accompanied by the standard error.
