# Supplementary material for: Acquisition of the physiological quality of peanut (Arachis hypogaea L.) seeds during maturation under the influence of the maternal environment
Source: PLoS One. 2021 May 3;16(5):e0250293. doi: 10.1371/journal.pone.0250293 (PMC8092650; doi:10.1371/journal.pone.0250293)
Supplement: S3 Table — Crop season 2017 and 2018. (DOCX) [file pone.0250293.s004.docx]

**S3 Table.** Statistical information on the observed data for peanut seed longevity, as assessed by p50 (sigmoid curves), days after flowering (DAF). Crop season 2017 and 2018.

| DAF | 2017 | 2018 |
| --- | --- | --- |
| 28 | 0 | 0 |
| 35 | 0 | 0 |
| 43 | 0 | 0 |
| 57 | 33.7 ± 1.1^*^ | 20.7 ± 0.4 |
| 76 | 41.3 ± 1.1 | 28.3 ± 0.6 |
| R^2^ | 0.99 | 0.99 |
| *p* *value* | <0.0001 | <0.0001 |

* The means of each variable are accompanied by the standard error.
